# Persistent Trigeminal Neuralgia‐Like Pain Following Isolated Hyaluronic Acid Lip Augmentation: A Case Report

**DOI:** 10.1111/jocd.70822

**Published:** 2026-04-01

**Authors:** Rocco Salvatore Calabrò, Piera Bonavita

**Affiliations:** ^1^ Spinal Cord Unit, IRCCS Centro Neurolesi “Bonino‐Pulejo” Messina Italy; ^2^ Otorhynolaringology Unit, “Giuseppe Fogliani” Hospital Milazzo Italy

## Abstract

**Background:**

Hyaluronic acid (HA) fillers are widely used for lip augmentation and are generally associated with a favorable safety profile. While vascular complications are well recognized, non‐vascular neurological adverse events, including trigeminal neuropathic pain, remain underreported, particularly following procedures in anatomically lower‐risk areas such as the lips.

**Objective:**

To report a rare case of persistent trigeminal neuralgia‐like pain following HA lip augmentation and to highlight diagnostic and therapeutic considerations.

**Methods:**

A 49‐year‐old woman developed persistent facial pain 10 days after HA lip augmentation. Clinical evaluation included dental assessment, imaging studies, and high‐resolution ultrasound of the perioral region. Initial management with hyaluronidase, corticosteroids, and non‐steroidal anti‐inflammatory drugs was performed. Due to persistent symptoms, a neuropathic pain‐oriented treatment with pregabalin and palmitoylethanolamide (PEA) was initiated. Pain severity was assessed using a visual analog scale (VAS), and follow‐up was conducted over 3 months.

**Results:**

The patient presented with severe bilateral facial pain and paresthesia (VAS 7), refractory to conventional anti‐inflammatory treatment. Imaging excluded dental pathology and vascular complications, while ultrasound revealed localized post‐procedural tissue changes. Following initiation of pregabalin and PEA, a significant and sustained reduction in pain was observed (VAS 2 at 1 month; VAS 1 at 3 months).

**Conclusion:**

Persistent trigeminal neuralgia‐like pain represents a rare but clinically relevant complication of HA lip augmentation. This case supports a neuropathic and neuroinflammatory mechanism and underscores the importance of early recognition and targeted treatment. Awareness of this complication may improve clinical outcomes and guide management strategies in aesthetic practice.

## 1 Introduction

Hyaluronic acid (HA) dermal fillers are among the most frequently performed minimally invasive aesthetic procedures worldwide, particularly for lip augmentation. They are generally considered safe and well‐tolerated, and most reported adverse events are mild, transient, and confined to the injection site, including edema, bruising, or asymmetry. Although uncommon, more severe complications, such as vascular occlusion, are well recognized and have been extensively described in the literature (Table 1) [1, 2].

**TABLE 1:** Overview of potential local, vascular, infectious, inflammatory, and neurological complications following facial dermal filler injections.

Category	Complication	Clinical features	Typical onset	Comments
Local/Mild	Pain, erythema, edema	Injection‐site discomfort, swelling, redness	Immediate–hours	Usually self‐limited
	Bruising/hematoma	Ecchymosis, tenderness	Immediate–days	Related to vessel puncture
	Asymmetry	Uneven volume or contour	Immediate–days	Often technique‐ or swelling‐related
	Overcorrection	Excess volume	Immediate	May require hyaluronidase (HA fillers)
Infectious	Local infection	Erythema, warmth, pain, purulent discharge	Days–weeks	Requires antibiotics
	Abscess	Fluctuant painful mass	Days–weeks	Rare; may need drainage
	Biofilm‐related infection	Indolent swelling, pain	Weeks–months	Often misdiagnosed
Inflammatory/Immune	Delayed inflammatory reaction	Swelling, erythema, tenderness	Weeks–months	May be immune‐mediated
	Granuloma formation	Firm nodules	Months–years	More common with non‐HA fillers
	Hypersensitivity reaction	Diffuse edema, erythema	Days–weeks	Rare with HA
Vascular	Intravascular injection	Severe pain, blanching	Immediate	Medical emergency
	Skin necrosis	Livedo, ulceration	Hours–days	Requires urgent treatment
	Visual loss/blindness	Sudden vision loss	Immediate	Rare but catastrophic
	Stroke	Neurological deficits	Immediate	Extremely rare
Neurological (Nonvascular)	Transient paresthesia	Numbness, tingling	Immediate–days	Often resolves spontaneously
	Nerve compression	Pain, sensory deficit	Days–weeks	Due to filler volume or edema
	Traumatic nerve injury	Persistent sensory changes	Immediate–weeks	Needle/cannula‐related
	Neuropathic pain/neuralgia‐like pain	Electric, stabbing, burning pain	Days–weeks	Rare; may persist
	Trigeminal neuropathy	Sensory loss or pain in the trigeminal territory	Days–months	Under‐recognized complication
Aesthetic/Functional	Tyndall effect	Bluish discoloration	Immediate	Superficial injection
	Migration of filler	Volume displacement	Weeks–months	Technique/product‐related
	Scarring	Fibrosis	Months	Rare
Systemic (rare)	Anaphylaxis	Hypotension, urticaria	Immediate	Extremely rare

Neurological complications after HA filler injections are rare and are most often reported in association with ischemic events or procedures performed in anatomically high‐risk facial areas. Trigeminal nerve involvement has been sporadically described, typically following injections in regions such as the nasolabial fold, cheek, or periorbital area, and in some cases in association with concurrent aesthetic procedures, including botulinum toxin administration [3]. From a diagnostic standpoint, however, it is important to distinguish classical trigeminal neuralgia from painful trigeminal neuropathy. According to ICHD‐3, classical trigeminal neuralgia is characterized by recurrent unilateral brief electric shock‐like pain, usually triggered by innocuous stimuli, whereas painful trigeminal neuropathy is more often associated with continuous or near‐continuous pain, sensory symptoms, and superimposed paroxysms. Painful post‐traumatic trigeminal neuropathy, when applicable, additionally requires a history of trauma to the trigeminal nerve and clinically detectable positive and/or negative sensory signs [4]. This distinction is particularly relevant when facial pain develops after a local cosmetic procedure and objective evidence of vascular compromise is absent.

By contrast, persistent neuropathic facial pain following isolated lip augmentation with HA is poorly documented. The perioral region is richly innervated by terminal branches of the trigeminal nerve, making it potentially susceptible to iatrogenic nerve irritation, local compression, or inflammatory reactions related to filler injection. In addition, delayed post‐procedural tissue reactivity may provide a biologically plausible explanation for symptoms that begin several days after treatment rather than immediately.

Here, we report the case of a 49‐year‐old woman who developed persistent trigeminal neuralgia‐like pain following HA filler injection limited exclusively to the lips. Because the clinical phenotype included bilateral pain, subjective paresthesia, and no demonstrable trigger zone, the presentation was interpreted more conservatively as procedure‐related secondary trigeminal neuropathic pain presenting with neuralgia‐like exacerbations rather than definite classical trigeminal neuralgia. The diagnostic work‐up included a dental assessment, perioral ultrasound, neurological evaluation, and a review of a recent brain MRI performed for chronic migraine follow‐up approximately 1 month before symptom onset. This wording is intentionally descriptive and conservative, avoiding overstatement of diagnostic certainty while still conveying the clinical phenotype. The pain was refractory to corticosteroids and nonsteroidal anti‐inflammatory drugs but responded to a neuropathic pain‐oriented therapeutic approach. This case highlights both a potentially under‐recognized complication of HA lip augmentation and the importance of precise diagnostic framing when post‐procedural facial pain mimics trigeminal neuralgia.

## 2 Case Description

A 49‐year‐old woman presented with persistent facial pain following HA lip augmentation. She had been undergoing aesthetic treatments on a regular basis for approximately three years, including facial botulinum toxin injections and nasolabial fold fillers, without any previous adverse events. No concomitant aesthetic procedures were performed at the session in which the lip filler was administered.

Her medical history was largely unremarkable, except for chronic migraine since early adulthood. For the past four years, she had been followed at a specialized headache center and treated with a monoclonal antibody targeting calcitonin gene‐related peptide (CGRP) (fremanezumab 225 mg administered monthly), with a good clinical response and no reported side effects. During particularly severe migraine attacks, she occasionally required short courses of corticosteroids or ketorolac. She denied any known drug or food allergies, apart from a single episode of adverse reaction to intramuscular ceftriaxone approximately 30 years earlier, characterized by transient edema of the tongue base. Importantly, the new facial pain was clearly distinct from her habitual migraine phenotype in quality, topography, and temporal pattern.

On November 7, 2025, the patient underwent elective lip augmentation using a BDDE‐crosslinked HA dermal filler intended for lip augmentation. The procedure was performed using a mixed technique, mainly with a blunt‐tip cannula and partially with a 30‐gauge needle. The injection was confined to the lips, through two entry points in the upper lip and two in the lower lip, with placement in the superficial lip soft tissues (vermilion/lip body) and a total injected volume of 0.6 mL distributed between the two lips. No filler was placed in the nasolabial folds, cheeks, chin, or other perioral aesthetic subunits. The procedure was uneventful, and no immediate complications were observed. Specifically, there was no severe immediate pain, blanching, livedoid discoloration, mucosal ischemia, or neurological symptoms noted during or immediately after the injection.

In the days following the treatment, the patient experienced mild lip swelling consistent with the expected post‐procedural course. Approximately ten days later, however, she developed new bilateral facial/oral pain, predominantly perceived along both dental arches, associated with tingling involving the palate and the tip of the tongue. She described the pain as sudden, stabbing/electric‐like episodes lasting several seconds up to approximately 1 min, recurring in short paroxysms superimposed on persistent dysesthesia, with a maximum intensity of 7 on the visual analogue scale (VAS). She did not identify a consistent trigger by chewing, talking, or light tactile stimulation. No fever, mucosal ulceration, skin necrosis, or other clinical signs suggestive of infection or vascular compromise were reported. Because of the close temporal relationship with the filler injection, treatment with hyaluronidase was performed. Systemic corticosteroids and ibuprofen were also prescribed, but these interventions provided little benefit.

**FIGURE 1:**
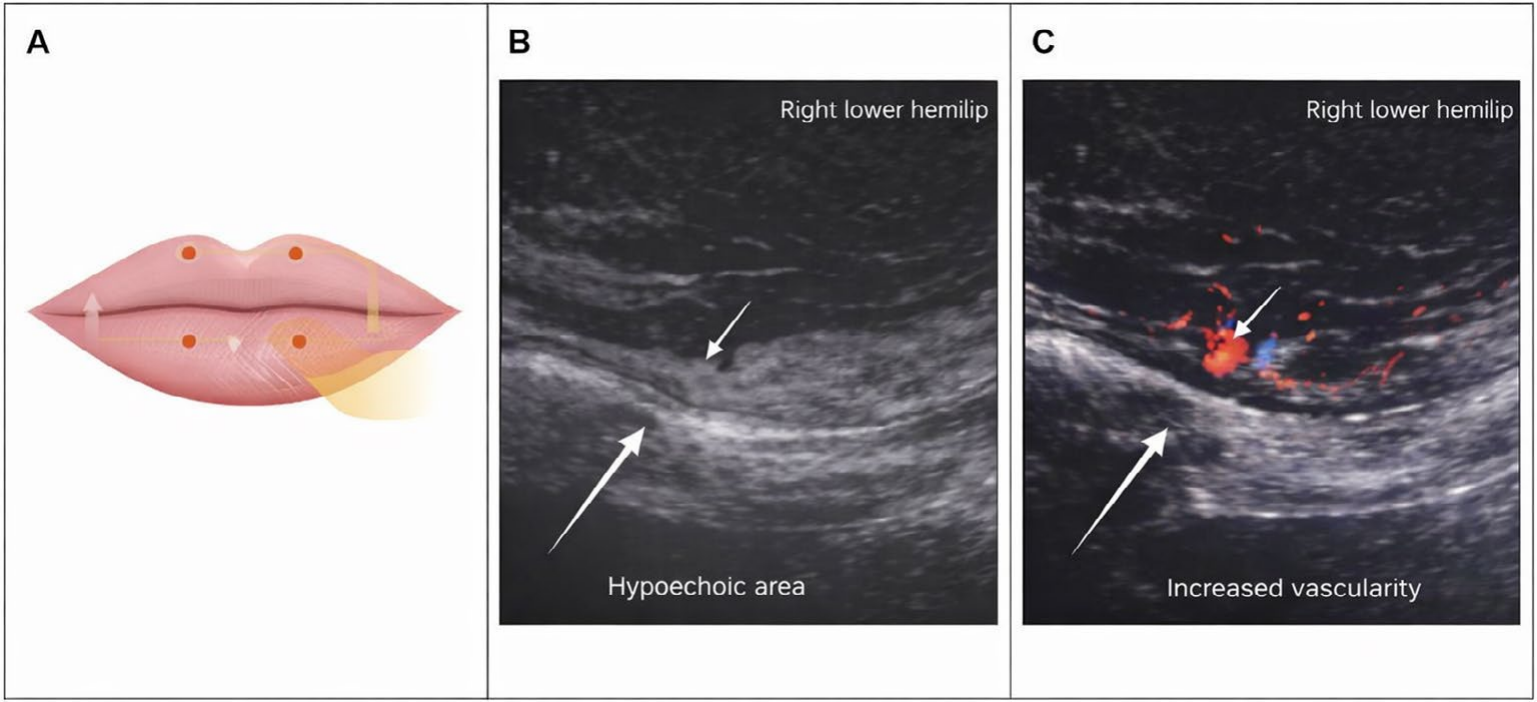
Injection‐site schematic and representative ultrasound findings. (A) Schematic diagram of the lip augmentation procedure showing the upper and lower lip entry points and the superficial vermilion/lip–body injection plane. (B) High‐resolution grayscale ultrasound of the right lower hemilip, just below the oral commissure, showing a small, poorly defined hypoechoic area (arrow). (C) Color Doppler image of the same region showing increased vascularity. In the clinical context, the finding was interpreted as localized post‐treatment tissue reactivity, with differential considerations including inflammatory reaction and/or a small residual filler deposit rather than definitive proof of the exact pain generator.

A dental evaluation, including radiographic imaging, did not reveal any abnormalities. In addition, a brain MRI performed approximately 1 month before the filler procedure as part of regular chronic migraine follow‐up was reviewed during the neurological work‐up; no demyelinating lesions, intracranial mass, skull base lesion, or other structural abnormalities capable of explaining the new facial pain had been reported. On December 1, 2025, a high‐resolution ultrasound of the perioral region was performed. This examination revealed a poorly defined hypoechoic area measuring approximately 7 x 2.5 mm in the right lower hemilip, just below the oral commissure, with increased vascularity on color Doppler imaging. In the clinical context, this finding was interpreted as a localized post‐treatment tissue change, with differential considerations including an inflammatory reaction and/or a small residual filler deposit; a granulomatous response was considered less likely because of the early timing, ill‐defined appearance, and absence of palpable nodules. The combination of indistinct margins and Doppler hypervascularity further favored reactive tissue change over a well‐circumscribed inert deposit, although imaging alone could not establish the exact pain generator. No fluid collections or foreign material were identified. Representative injection‐site schematic and ultrasound findings are shown in Figure 1.

Over the following weeks, the patient was treated with bromelain, intravenous thioctic acid (600 mg), and oral acetyl‐L‐carnitine (500 mg), without meaningful improvement. As symptoms persisted for nearly three months, she was referred for neurological evaluation. Neurological examination showed normal mental status and intact cranial nerves. Facial sensation to light touch and pinprick was symmetric in the V1, V2, and V3 territories bilaterally; corneal reflexes were preserved; masseter strength was normal; and no facial weakness was present. No objective hypoesthesia, allodynia, or hyperalgesia could be elicited, and no reproducible trigger point or trigger zone was identified on gentle stimulation of the perioral region or oral mucosa. Taken together, the bilateral distribution, subjective paresthesia, absence of objective neurological deficits, and close temporal relationship with the filler procedure supported a working diagnosis of secondary trigeminal neuropathic pain presenting with trigeminal neuralgia‐like paroxysms, rather than definite classical trigeminal neuralgia according to ICHD‐3 criteria. Treatment with pregabalin (150 mg/day) in combination with palmitoylethanolamide (PEA, 1400 mg/day) was initiated.

After approximately one month of this regimen, the patient reported a clear and sustained improvement in pain intensity and frequency (VAS 2). The paroxysmal component became infrequent, and the background dysesthesia markedly diminished. At the subsequent 3 month follow‐up, symptoms remained significantly reduced (VAS 1), with no clinical evidence of vascular complications, infection, or palpable filler‐related nodules.

## 3 Discussion

Neurological adverse events following HA dermal filler injections are considered uncommon and are most often described in association with vascular compromise or injections performed in anatomically high‐risk facial regions [1, 2, 3]. Nevertheless, nonvascular neurological complications, such as persistent sensory disturbances or neuropathic pain, are increasingly recognized in clinical practice, although they remain underreported, particularly after procedures generally perceived as low risk, including lip augmentation. Our case is noteworthy because the filler was injected exclusively into the lips; no concurrent botulinum toxin or filler procedure was performed, and no signs of ischemic injury were documented.

A central issue raised by this case is diagnostic terminology. The patient did not fully meet ICHD‐3 criteria for classical trigeminal neuralgia, which require recurrent unilateral brief electric shock‐like pain typically precipitated by innocuous stimuli. Nor could painful post‐traumatic trigeminal neuropathy be diagnosed with full certainty, because objective positive or negative trigeminal sensory signs were not demonstrable on neurological examination [4]. In contrast, her symptoms were bilateral, accompanied by subjective paresthesia involving the palate and tongue tip, and were not associated with a reproducible trigger zone. For this reason, we intentionally use the descriptive expression “trigeminal neuralgia‐like pain” and interpret the syndrome within a broader framework of secondary trigeminal neuropathic pain temporally related to a local perioral procedure. This wording intentionally avoids overdiagnosing classical trigeminal neuralgia while still capturing the clinically meaningful neuralgia‐like quality of the attacks.

The neuroimaging issue also deserves clarification. Current guidelines recommend MRI as part of the work‐up in patients with trigeminal neuralgia‐like symptoms because clinical features alone cannot reliably exclude secondary causes [5]. In the present case, a brain MRI had been obtained approximately 1 month before the cosmetic procedure during routine migraine follow‐up and did not show structural abnormalities that would account for the subsequent facial pain. Although this recent negative MRI reduced the likelihood of central secondary causes such as demyelinating disease or space‐occupying lesions, we acknowledge that it was not a dedicated post‐onset trigeminal MRI protocol. This should therefore be regarded as a limitation of the case rather than definitive proof of peripheral causation. Thus, the recent MRI serves as an important exclusionary element but not as a substitute for dedicated post‐onset trigeminal imaging.

The approximately 10‐day delay between injection and symptom onset also argues against an immediate direct nerve transection as the sole mechanism. A more plausible explanation is an evolving local process, such as delayed tissue edema, perineural compression, or localized neuroinflammatory reaction in the densely innervated perioral soft tissues. The ultrasound finding of a small ill‐defined hypoechoic hypervascular area supports the presence of a persistent local post‐treatment reaction, but it does not by itself establish a direct anatomical correlation with the patient's bilateral pain. Accordingly, the causal link in this case rests on temporality, anatomical plausibility, exclusion of major alternative causes, and therapeutic response, rather than on definitive mechanistic proof. The limited response to hyaluronidase may further suggest that once symptoms became clinically manifest, the dominant process was no longer simple filler bulk alone but secondary perineural/inflammatory sensitization [2, 6, 7].

The sonographic abnormality should be interpreted cautiously. In our view, the main differential diagnoses are a localized inflammatory reaction and a small residual filler deposit, whereas a granulomatous response appears less likely given the early presentation, lack of a well‐defined nodule, and subsequent clinical improvement without surgical intervention. The fact that the ultrasound abnormality was unilateral whereas symptoms were bilateral further suggests that ultrasound served chiefly to document ongoing local tissue reactivity rather than to pinpoint the exact pain generator. Conversely, the absence of a discrete encapsulated nodule, abscess, or collection makes a mature granulomatous or infectious process less compelling at this stage.

Beyond mechanical factors, a prolonged local neuroinflammatory response may also have played a role. HA fillers, while generally biocompatible, can trigger localized immune and inflammatory reactions that, in susceptible individuals, may promote peripheral nerve sensitization. If such processes persist, they may contribute to central sensitization and pain chronicity [2, 6, 7]. This hypothesis may be especially relevant in patients with a pre‐existing pain disorder, such as migraine, even when the new pain is clinically distinct from the baseline headache phenotype.

The patient's favorable response to pregabalin in combination with palmitoylethanolamide (PEA) further supports the neuropathic and neuroinflammatory nature of the pain. Pregabalin is a well‐established treatment for neuropathic pain and has been used in trigeminal neuropathic conditions [5, 8]. In this case, the lack of meaningful benefit from corticosteroids, ibuprofen, bromelain, thioctic acid, and acetyl‐L‐carnitine contrasted with the clear improvement observed after the introduction of pregabalin plus PEA, reinforcing a neuropathic pain model over a purely inflammatory or odontogenic process.

PEA, an endogenous fatty acid amide, has demonstrated anti‐inflammatory, analgesic, and neuroprotective effects, mediated in part by modulation of mast cell activation and glial reactivity [6, 7]. Clinical evidence supports its use in various neuropathic pain syndromes, including entrapment neuropathies such as pudendal neuralgia [9]. Emerging data also suggest a role for PEA in trigeminal neuropathic pain and painful post‐traumatic trigeminal neuropathy [10, 11]. In this context, the combination of pregabalin and PEA represents a rational, mechanism‐based therapeutic strategy targeting both neuronal hyperexcitability and the underlying neuroinflammatory milieu.

Several limitations should be acknowledged. This is a single case report; no dedicated post‐onset trigeminal MRI, quantitative sensory testing, or electrophysiological assessment was available, and the proposed mechanism remains inferential. Nonetheless, the temporal association with an isolated lip procedure, the exclusion of odontogenic and major structural intracranial causes, the localized ultrasound abnormality, and the response to neuropathic pain‐oriented therapy make a filler‐related peripheral neuropathic complication a clinically plausible interpretation.

## 4 Clinical Implications for Aesthetic Practice

Although rare, neuropathic pain should be suspected when patients report electric, stabbing, or burning facial/oral pain persisting beyond the typical recovery period after lip augmentation. Particular caution is warranted when pain is perceived as bilateral dental arch pain, because this phenotype may initially be misattributed to an odontogenic disorder despite a negative dental assessment. A structured reassessment should document pain phenotype, laterality, sensory symptoms, and the presence or absence of trigger zones or neurological deficits. Early reassessment is recommended to exclude vascular or infectious complications. Ultrasound examination may assist in evaluating local tissue changes. When symptoms are atypical for classical trigeminal neuralgia, recent neuroimaging and/or dedicated MRI should be reviewed or obtained as clinically indicated. From a preventive perspective, a careful knowledge of perioral anatomy, avoidance of excessive filler volume in confined spaces, minimization of repeated needle passes, and gentle injection technique may help reduce the risk of nerve irritation. Prompt recognition and timely referral for appropriate management may reduce the likelihood of chronic pain and improve patient outcomes.

## 5 Conclusions

This case illustrates a plausible but not definitively proven neurological complication of HA lip augmentation, namely persistent trigeminal neuralgia‐like pain occurring in the absence of vascular adverse events. More precisely, the clinical presentation is best framed as secondary trigeminal neuropathic pain with neuralgia‐like paroxysms after an isolated lip filler procedure. Terminological precision is essential; describing such cases simply as trigeminal neuralgia may imply a level of diagnostic certainty not supported by the available findings. Although lip augmentation is generally considered a low‐risk aesthetic procedure, the dense trigeminal innervation of the perioral region may predispose susceptible individuals to iatrogenic nerve irritation and neuropathic pain. The failure of conventional anti‐inflammatory treatments and the subsequent response to pregabalin combined with palmitoylethanolamide support a predominantly neuropathic and neuroinflammatory mechanism. Awareness of the diagnostic distinction between classical trigeminal neuralgia and post‐procedural trigeminal neuropathic pain is essential to avoid overclassification and to guide appropriate work‐up and treatment. Greater awareness of this under‐recognized complication may help improve diagnostic accuracy and management strategies following perioral filler injections.

## Funding

This work was supported by Italian Ministry of health.

## Ethics Statement

Ethical approval was not required for this single case report according to institutional policies, as it deals with an adverse event following a normal procedure. The patient gave consent for the publication of the case.

## Consent

Written informed consent was obtained from the patient for publication of this case report and any accompanying clinical information.

## Conflicts of Interest

The authors declare no conflicts of interest.

## Data Availability

The data that support the findings of this study are available on request from the corresponding author. The data are not publicly available due to privacy or ethical restrictions.
